# Neuroglobin, a Factor Playing for Nerve Cell Survival

**DOI:** 10.3390/ijms17111817

**Published:** 2016-10-31

**Authors:** Diego Guidolin, Cinzia Tortorella, Manuela Marcoli, Guido Maura, Luigi F. Agnati

**Affiliations:** 1 Department of Neuroscience, University of Padova, Padova 35122, Italy; cinzia.tortorella@unipd.it; 2 Department of Pharmacy and Center of Excellence for Biomedical Research, University of Genova, Genova 16126, Italy; marcoli@pharmatox.unige.it (M.M.); maura@pharmatox.unige.it (G.M.); 3 Department of Biomedical Sciences, University of Modena and Reggio Emilia, Modena 41121, Italy; luigi.agnati@gmail.com; 4 Department of Neuroscience, Karolinska Institutet, Stockholm 17177, Sweden

**Keywords:** apoptosis, neuroglobin, mitochondria, cytochrome *c*, protein–protein interaction

## Abstract

Cell death represents the final outcome of several pathological conditions of the central nervous system and available evidence suggests that in both acute injuries and neurodegenerative diseases it is often associated with mitochondrial dysfunction. Thus, the possibility to prevent mitochondrial events involved in cell death might represent efficient tools to limit neuronal damage. In recent years, increased attention has been paid to the endogenous protein neuroglobin, since accumulating evidence showed that its high expression was associated with preserved mitochondrial function and to an increased survival of nerve cells in vitro and in vivo in a variety of experimental models of cell insult. The biological and structural features of neuroglobin and the mitochondria-related mechanisms of neuroglobin-induced neuroprotection will be here briefly discussed. In this respect, the inhibition of the intrinsic pathway of apoptosis emerges as a key neuroprotective effect induced by the protein. These findings could open the possibility to develop efficient neuroglobin-mediated therapeutic strategies aimed at minimizing the neuronal cell death occurring in impacting neurological pathologies like stroke and neurodegenerative diseases.

## 1. Introduction

Cell death represents the final outcome of several pathological conditions of the central nervous system (CNS), including acute neurological disorders (such as cerebrovascular or traumatic events) and neurodegenerative diseases (such as Alzheimer’s disease, Parkinson’s disease, amyotrophic lateral sclerosis and Huntington’s disease). The localization and the extent of the insult define the gravity of the resulting neurological defect, which can also lead to loss of life or to severe long-term disability.

Damaged neurons and glial cells can follow different death pathways, ranging from necrosis to apoptosis (see [1]), depending on both internal (e.g., physiologic milieu, metabolic status or cell type) and external (e.g., type, intensity and duration of the injury) features [2,3,4]. Several lines of evidence, however, suggest that, irrespective of the type of its manifestation, cell death that occurs in the CNS in both acute injuries and neurodegenerative diseases is often associated with mitochondrial dysfunctions [4,5]. In fact, in many cases, both necrosis and apoptosis processes share a starting step (see [6] for a detailed review) in which physiological and pathological stimuli (such as DNA damage, low nutrient levels, increased calcium levels, receptor signaling, oxidative stress and intracellular aggregation of misfolded proteins) trigger a change in the inner mitochondrial membrane that results in the opening of the mitochondrial permeability transition pore [7], a dynamic multiprotein complex (involving the voltage-dependent anion channel (VDAC)) formed in a contact site between the inner and outer mitochondrial membranes, with release of mitochondrial factors in the cytosol. The increase in mitochondrial membrane permeability can lead to collapse of the mitochondrial inner transmembrane potential, uncoupling of the respiratory chain, hyper production of superoxide anions, disruption of mitochondrial biogenesis, outflow of matrix calcium and glutathione, and release of soluble intermembrane proteins. Since neurons are particularly dependent on mitochondria because of their high energy demand [8], the consequent mitochondrial dysfunction can entail a true bioenergetics catastrophe culminating in the disruption of plasma membrane integrity with cell necrosis. Alternatively, the increased mitochondrial membrane permeability can trigger the mitochondrial-dependent (intrinsic) pathway of apoptosis (see [9] for a review) in which the released cytochrome *c* (Cyt-C) binds the apoptotic protease-activating factor 1 (APAF1), leading to the formation of a caspase activation platform (apoptosome). The apoptosome recruits and activates an initiator caspase (caspase-9), which, in turn, cleaves and activates the execution caspases (such as caspase-3 and caspase-7), leading to cytoskeletal reorganization and disintegration of the cell into apoptotic bodies. Additional proteins, however, such as those from the Bcl2 family [10] and IAP (inhibitors of apoptosis proteins) family [11], can interfere with the pathway of apoptosis and the cell only commit to death if stress signals overcome all the protective measures. The relative rate of the two mentioned processes (bioenergetic catastrophe versus protease and endonuclease activation) determines whether a cell will undergo primary necrosis or apoptosis [6].

The fact that in the CNS mitochondrial events play a key role in controlling cell death has major implications for the development of neuroprotective treatments. In fact, agents preventing mitochondrial membrane permeabilization or inhibiting post-mitochondrial executioners of cell death might represent efficient tools to limit neuronal damage. In particular, the possible stimulation of endogenous neuroprotective mechanisms has emerged as a promising strategy [5]. In this context, in the last years, increased attention has been paid to neuroglobin (NGB), an oxygen-binding globin that has been demonstrated to be an endogenous neuroprotective molecule likely related to mitochondrial function and regulation [12]. In the present review article, available evidence on NGB and on the possible mechanisms of action involved in NGB’s neuroprotection, with particular reference to its anti-apoptotic role, will be briefly summarized and discussed.

## 2. Neuroglobin

### 2.1. Structure and Expression

NGB is an oxygen-binding globin protein whose presence in the nerve tissues was demonstrated in 2000 by Burmester and coworkers [13] following a bioinformatics analysis of the just published human genome.

**Figure 1:**
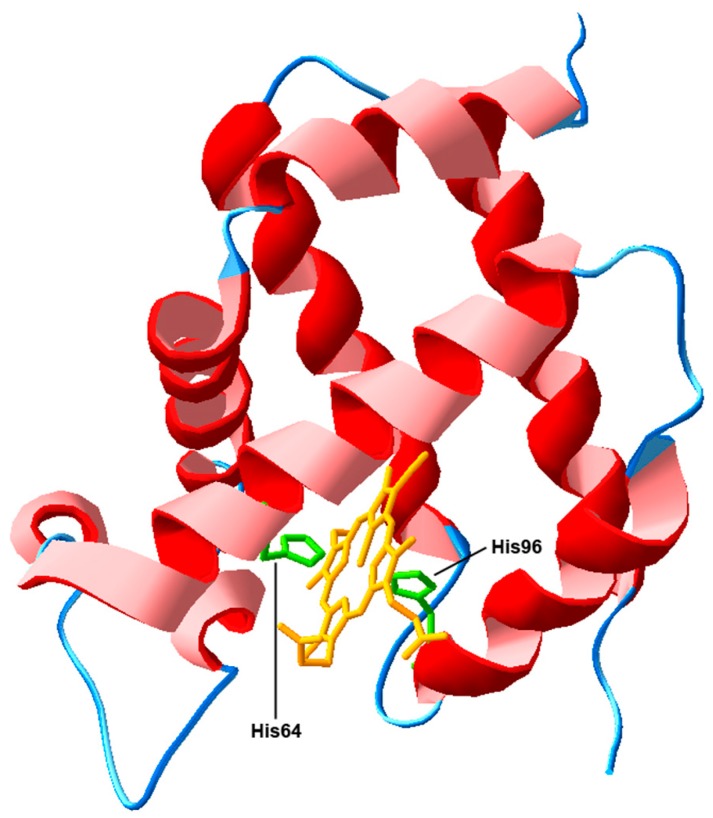
3D protein structure of human neuroglobin (NGB; PDB code: 1OJ6) exhibiting the typical globin fold (see [18]). In green are represented the two histidine side chains (His64 and His96) interacting with the heme group represented in yellow. The most peculiar structural characteristic of NGB is the so-called six-coordinate binding scheme of the heme Fe atom. In the absence of external ligands, the His64 binds the heme iron at its sixth, distal position. Thereby, any external gaseous ligand such as O_2_ or NO has to compete with the internal His64 ligand for Fe binding [19].

Structural analyses [14,15] have indicated that human NGB displays the typical globin fold (Figure 1), comprised of 151 amino acids (molecular mass, 17 kDa), with only 20%–25% of sequence identity with myoglobin and hemoglobin. It contains a single noncovalently bound protoheme IX prosthetic group that, unlike the other vertebrate globins, is predominantly six-coordinate with the heme iron atom binding two histidine side chains. This structure, however, is in equilibrium with a small amount of a mono-histidine (five-coordinate) form, representing the structure reactive with the typical gaseous heme ligands [16,17].

As far as the distribution of the protein in the CNS is concerned, available data indicate a significant presence in a relatively limited number of areas (see Table 1 for a summary) of both mouse [20,21] and human CNS [22]. They appear to correspond to metabolically active, oxygen consuming cell populations [23,24], as exemplified by retinal cells [25]. NGB was mainly (~90%) localized in the cytosol [26], but accumulating evidence revealed that it is also associated with mitochondria, as demonstrated by immunohistochemistry [27], yeast two hybrid assays [28] and biochemical studies [29].

NGB is a particularly highly conserved protein, with mouse [30] and human NGB differing in only 6% of the amino acid positions and it has a substitution rate almost four-fold lower than that of other vertebrate globins [18], suggesting that its functions are of basic importance to some types of tissues.

### 2.2. Neuroprotective Role

The neuroprotective role of NGB in a wide range of pathological conditions has been well documented by a quite large number of experimental studies.

In cultured cortical neurons, the antisense-mediated knockdown of the globin rendered the cells more vulnerable to hypoxia [31] and decreased viability in neuroblastoma cells under oxidative stress [32]. Conversely, when cultured cortical neurons were exposed to oxygen-glucose deprivation and reoxygenation (OGD/R) to induce neuronal damage, the combined treatment with mild hypothermia and NGB significantly increased cell viability [33,34], confirming the protective effect of the protein against OGD/R-induced neuronal injury. These results are in line with previous data showing that, in cultured cells, overexpression of NGB protected against hydrogen peroxide [35]. Cerebellar granule neurons were recently used [36,37] to study NGB in relation to neurotoxic challenges induced by sodium arsenite (NaAsO_2_). RNA interference technology was used to silence NGB and the results indicated that in silenced cells the NaAsO_2_-induced cytotoxicity was exacerbated, suggesting a protective role for NGB in this pathological condition. Amyloid beta (Aβ)-induced cell death was also counteracted by NGB overexpression by plasmid (pcDNA3-Ngb) transfection, as demonstrated by studies on PC12 cells [38] and, more recently, NGB has been shown to interact with heme-Aβ complexes known to catalyze oxidation of neurotransmitters and to have been associated with Alzheimer’s disease [39].

In vivo studies provided further support to the neuroprotective role of the globin. In NGB-transgenic mice, NGB overexpression with more than 2.7-fold protein level increase driven by CMV promoter [40] or by chicken β-actin promoter [41] ameliorated the severity of histological and functional deficits in stroke models. Moreover, in the classical model of focal cerebral ischemia in rats, the combined treatment of exogenous NGB and hemin was shown to induce a significant improvement in neurobehavioral scores, brain water content and infarct volume ratio [42]. Consistently, knockdown of endogenous NGB by gene silencing worsened the outcome of focal cerebral ischemia in rats [43]. In a rat model of cardiac arrest and resuscitation, Fan and coworkers [44] recently found that remote ischemic preconditioning significantly decreased the occurrence of neuronal apoptosis and necrosis. The effect was associated with an increased NGB expression 24 h after return of spontaneous circulation. Furthermore, following administration of antisense NGB nucleotides before induction of remote ischemic preconditioning overexpression of the globin was decreased and, correspondently, the neuroprotective effect was partly abrogated. NGB overexpression resulted also protective against traumatic brain injury [45,46] and β-amyloid-induced neurotoxicity and Alzheimer phenotype in NGB and APP (amyloid precursor protein) double-transgenic mice [47]. Furthermore, in vivo studies on retinal damage [48,49] and ischemia [50] demonstrated that NGB overexpression protected retinal ganglion cells. Whereas the majority of the studies indicated a protective effect of NGB, data raising some question on the capacity of NGB to provide general protection to neurons in vivo were also reported. In NGB-null mice undergoing permanent middle cerebral artery occlusion, for instance, the lack of NGB did not affect neuronal and organismal survival rate, although it altered the hypoxia-induced c-FOS and HiflA gene expression and the regulation of the glycolythic pathway genes [51]. Moreover, 24 h after the ischemic insult a smaller infarct size was observed in NGB-null mice as compared to wild type controls [52,53]. Although one cannot exclude that inborn deficiency in NGB function might induce compensatory mechanisms, these studies suggested that, at least at the endogenous expression levels, NGB is not protective against ischemic insult. Probably it is a stress-inducible protein that could protect nerve cells against injuring stimuli when expressed at high levels [54].

Further evidence comes from human population studies, that have associated genetic polymorphisms within the human neuroglobin gene with neuroprotection, while decreased expression of neuroglobin in older people, in women, or associated with single nucleotide polymorphism has been linked to increased risk of Alzheimer’s disease [55,56,57].

## 3. Possible Mechanisms of NGB Neuroprotection

As pointed out by Brittain et al. and Yu et al. [12,58], in many of the above mentioned studies, where NGB exhibited a significant effect on nerve cell survival, parameters linked to mitochondrial functions were significantly affected by NGB expression. They include ATP production [45] and reactive oxygen species (ROS) generation [34,39,48]. NGB treatment was able to stabilize mitochondrial membrane potential [34] and NGB overexpression was associated with reduced mitochondrial DNA damage [50]. Furthermore, as a consequence of NGB overexpression, most of the studies reported a significant decrease of death signaling and a downregulation of molecules involved in the apoptotic cascade [32,36,37,42,44,45,50].

Data also exist indicating a potential involvement of NGB in mitochondrial dynamics. Mitochondria, in fact, are very dynamic organelles undergoing fission and fusion to interchange their contents and actively transported to subcellular sites where energy is required. NGB overexpression can eliminate hypoxia-induced aggregation of the organelles [59] and it has been suggested that NGB may also play a role in mitochondrial transportation ameliorating H_2_O_2_-induced actin condensation [60].

**Figure 2:**
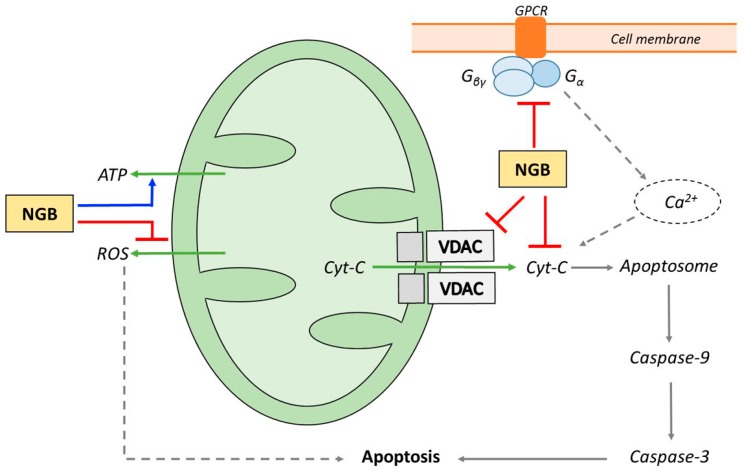
Simplified scheme illustrating the potential mechanisms of neuroglobin (NGB) neuroprotection modulating mitochondria. On the left, actions based on the interaction with heme-iron ligands are indicated. They involve preserving mitochondrial ATP production and scavenging of ROS (see [12]). On the right, processes based on the interaction with proteins involved in the regulation of the intrinsic pathway of apoptosis are reported. They include direct interactions with G proteins [61], voltage-dependent anion channel (VDAC) [62] and especially with cytochrome *c* (Cyt-C) [63]. The whole pattern of reactivity can contribute to the inhibition of the apoptotic process (see text). Blue arrows emphasize stimulatory and red lines inhibitory NGB actions. Solid arrows illustrate the steps of the intrinsic pathway of apoptosis. Other relevant processes are indicated by dashed arrows.

These findings, together with evidence showing that NGB expression is basically confined to metabolically active, oxygen-consuming cell types (see Section 2.1), led to the hypothesis that mechanisms triggered by NGB could induce neuroprotection by modulating mitochondria function and regulation (see [12,58] for reviews). The exact processes, however, are still under careful investigation. Basically, two mechanisms of action have been considered. They are summarized in Figure 2 and will be briefly discussed in the sections that follow.

### 3.1. Actions Based on the Interaction with Heme-Iron Ligands

Although NGB is an O_2_-binding protein, the initial hypothesis that its protective role was to facilitate oxygen diffusion during periods of anoxia [25] has been challenged (see [58,64]) due to the low levels of the protein in the brain (<1 µM) and to the high O_2_ binding rate but low O_2_ dissociation rate of NGB [65,66,67]. However, although the biophysical properties of NGB are not optimal for oxygen transport, it has been suggested that the protein could actually play this physiological role at least in some district, as, for instance, in the retina [68]. It has also been proposed (see [12]) that the O_2_-binding properties of NGB could imply a role for the protein in O_2_ sensing and energy metabolism, i.e., ATP production. This hypothesis found some support on experimental data showing that the decline of ATP levels after hypoxia/reoxygenation was significantly ameliorated by NGB overexpression [69,70]. It is still undefined, however, whether such a preservation of mitochondrial ATP production by NGB occurred through preserving the general mitochondrial function or by a specific influence on mitochondrial respiration.

Studies using recombinant human NGB confirmed that the protein can efficiently scavenge a variety of ROS, including superoxide anion, hydrogen peroxide, and hydroxyl radical [71]. The reaction with nitric oxide (NO) is a further reactivity that NGB shares with myoglobin [72]. Based on this characteristic was the suggestion of a role for NGB in the protection against NO- and reactive nitrogen species (RNS)-induced neurotoxicity [73] as a mechanism of NGB neuroprotection. Actually, in a mouse model of ischemia-reperfusion injury NGB overexpression was associated with a significant reduction of ROS/RNS production and lipid peroxidation in the CA1 region of the hippocampus [74]. In this respect, it has to be pointed out that globins can eliminate NO by expressing NO dioxygenase activity. In the NO deoxygenation reaction, the ferrous oxy-heme complex reacts with NO to form ferric heme and nitrate [75]. However, since NGB exhibits a relatively rapid autoxidation and in vivo is expressed in quite low amount, the presence of a reductant or enzymatic reducing system to recover the ferrous form would be needed in order to achieve an efficient elimination of NO [58,76,77]. Despite considerable efforts, such a re-reducing system has never been identified in vivo [78,79,80,81]. Abbruzzetti and coworkers [82] described the presence in the NGB molecule of multiple binding sites allowing temporary docking of small gaseous ligands for relatively long times (up to several hundred microseconds). The authors suggested that this feature could be consistent with a catalytic role of NGB in detoxification of NO by way of a NO dioxygenase reaction, since it would facilitate the sequential reaction of substrates with the heme. Of particular interest, however, is a recent work from Liu and Brittain [83] based on a mathematical model of the steady state concentration of ferrous form of NGB under various conditions. The analysis indicated that despite the fast rates of autoxidation of NGB, low concentrations of NGB in a reduced state could actually be maintained in vivo.

Summing up, although the reactivity features of NGB with heme-iron ligands can have pleiotropic effects not restricted to mitochondria, they can influence their function (e.g., in terms of ATP and ROS/RNS production [12]) and may represent an important component of the processes driving NGB-induced neuroprotection, in particular when protein overexpression occurs.

### 3.2. Structural Biology of NGB and Protein-Protein Interactions

In addition to heme ligands reactivity, other interactions have been investigated to further explore the NGB-induced protection from cell death. In this respect, a bioinformatics analysis [84] aimed at characterizing the propensity of NGB to establish protein–protein interactions showed that more than 80% of the amino acids at the molecule surface have non-null propensity, suggesting that NGB could, at least in theory, exploit a number of different sites for interaction with other proteins. Furthermore, a search in the STRING database [84,85] for known and predicted protein–protein interactions involving human NGB, indicated the possibility for NGB to interact with a number of proteins involved in the regulation of the apoptotic process, such as Cyt-C, VDAC and elements of signal transduction mechanisms (such as G proteins). Most of the indicated interactions were predicted by STRING as direct interactions, involving the binding of the proteins to NGB.

As a matter of fact, for what it concerns Cyt-C, a very rapid reaction between ferrous NGB and ferric Cyt-C was demonstrated [63]. Furthermore, the reaction, like other characterized protein–protein redox reactions, occurred via an intermediate protein–protein complex with an equilibrium dissociation constant in the range of 200 µM, as demonstrated by Surface Plasmon Resonance [86].

**Figure 3:**
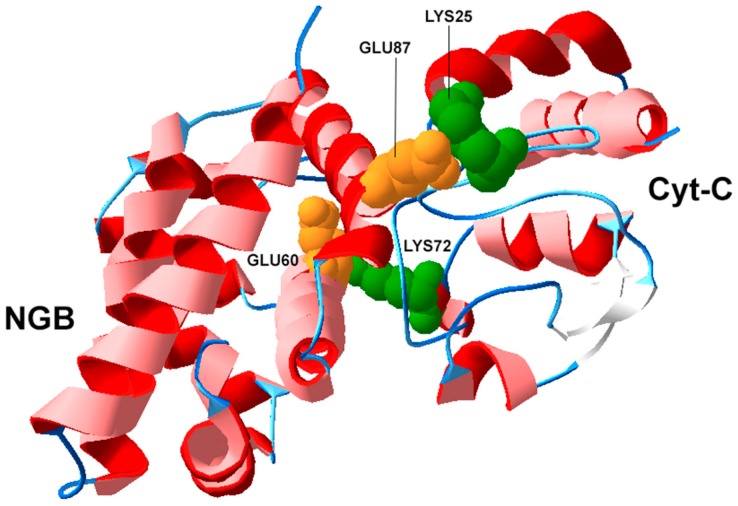
Putative structure predicted by docking studies [84] between human neuroglobin (NGB; PDB code: 1OJ6) and human cytochrome *c* (Cyt-C; PDB code: 3NWV). Relevant amino acids at the interaction interface are highlighted in color. Interface residues Glu60 and Glu87 on NGB are shown in yellow, the corresponding residues on Cyt-C (Lys72 and Lys25, respectively) in green.

In the absence of direct structural determinations of the complex formed in the reaction of NGB with Cyt-C, computational methods (and in particular docking methods) have been used to gain further insights [84,87]. An interesting finding (see Figure 3) was that the interface residues Glu60 and Glu87 on NGB bind residues, Lys72 and Lys25, on Cyt-C, that are mandatory for the binding of Cyt-C to APAF1, a key apoptosome forming protein [87,88]. Thus far, however, these amino acid interactions have not been experimentally validated.

Another protein–protein interaction involving NGB has been suggested by experimental data showing that the globin modulates GPCR signaling [89] by interacting with the G_α_ subunits of the trimeric G protein. The possible docking between NGB and G_α_(i) has been analyzed [84] and the residues crucial for the interaction experimentally identified [90]. Interestingly, the G_α_(i) molecular regions involved in the predicted interface appear available when the molecule is complexed in the GPCR [91], a result in line with available experimental data showing that NGB binds to the GDP-bound state of the G protein α subunit, and inhibits GDP dissociation [61], thereby protecting neuronal cells against oxidative stress [52,92] and inhibiting calcium rise in the cytosol that in turn can activate apoptosis [58,93].

A further intriguing suggestion from docking studies (see [84]) concerned the possible interaction between NGB and VDAC. In particular, the predicted configuration of the complex NGB-VDAC involved the formation of an interface at the level of the channel aperture, opening the possibility for NGB to directly modulate the permeability of the outer mitochondrial membrane [94,95] and the release of Cyt-C, which represents the key event leading to apoptotic cell death [9]. Experimental data consistent with this view have been provided in primary cultured mouse cortical neurons following oxygen-glucose deprivation (OGD) [62]. The results indicated that the binding between NGB and VDAC was increased after OGD compared to normoxia. Furthermore, NGB overexpression significantly reduced OGD-induced mitochondria permeability transition pore opening markers.

For what it concerns the mechanisms described in Section 3.1 and Section 3.2, a first point to emphasize is that they are closely related. The redox and ligation state of the heme-iron, for instance, are important for the mechanisms involving the interaction between NGB and Cyt-C, since the presence of reduced NGB is pivotal to obtain the reduction of oxidized Cyt-C. Furthermore, considering the above described pattern of NGB interactions as a whole, the hypothesis that neuroprotection by NGB could involve a significant modulation of the intrinsic pathway of apoptosis can be put forward [58,76,87]. This point will be the focus of the next section.

## 4. NGB and Apoptosis

The role of NGB as ROS scavenger and in the protection against NO- and RNS-induced neurotoxicity [73] could enable the cell to reduce the amount of intracellular toxic stimuli and prevent apoptosis [96,97]. Furthermore, NGB can modulate the apoptotic process by exploiting direct interactions with proteins such as VDAC [9], G_α_ proteins [92] and Cyt-C [58]. Of particular relevance in this context is the role it plays in reducing released Cyt-C to the inactive ferrous form [63]. Monte Carlo computational modeling of the apoptotic process, based on measured reaction rate constants, where diffusion and reaction of signaling molecules are simulated at an individual molecular level, were used to probe the effects of variations in protein concentrations and the significance of the reactivity with Cyt-C [98]. This approach allowed a deeper characterization of the set of reactions influenced by NGB and the hypothesis was broadened to suggest a coordinating role (going beyond its simple redox reaction) for the protein in the mitochondrial-dependent pathway of apoptosis. In particular, it has been suggested that cell protection can be achieved when the NGB/Cyt-C ratio is at least 3/1 indicating that the NGB/Cyt-C ratio may represent a parameter that determines the cell fate [67,87]. Thus, NGB rather than simply preventing the apoptotic process would reset the level of cellular insult required to trigger the apoptotic cascade (see [58,76]).

Several lines of experimental evidence consistent with these views have been accumulated, providing a correlation between NGB expression levels and a number of markers of apoptosis.

The ability of NGB to interfere with the mitochondrial-dependent pathway of apoptosis and, in particular, to interact with Cyt-C has been directly investigated in several in vitro studies. In cell lysates devoided of mitochondria, but containing APAF1 the pro-apoptotic activity of Cyt-C was lost in the presence of NGB [87] Consistently, in neuronal cells under hypoxic stress a regulation of Cyt-C release by NGB has been reported [99]. In neuroblastoma cells undergoing H_2_O_2_-induced apoptosis the upregulation of NGB by 17β-estradiol strongly increased the association between NGB and Cyt-C into the mitochondria and reduced Cyt-C and caspase-3 levels in the cytosol, demonstrating that the prevention of Cyt-C release is one of the pivotal mechanisms underlying NGB neuroprotection [100].

As far as signals downstream to Cyt-C release are concerned, increased NGB expression in primary cortical neurons obtained by using a transactivator-of-transcription protein-transduction domain-neuroglobin fusion protein (TAT PTD-Ngb) decreased the activity of caspase-3 and caspase-9 in response to hypoxia [101,102]. Suppression of the activity of caspase-3 and caspase-9 by NGB overexpression was also observed in human neuroblastoma SH-SY5Y cells undergoing BH3 mimetic-induced apoptosis [103] or amyloid-β toxicity [104].

In the in vivo study by Lan and coworkers [105] NGB overexpression was induced in rats by adeno-associated virus injection seven days before spinal cord injury (SCI). Histopathological and biochemical evaluations were performed 24 h after SCI. The results showed that animals overexpressing NGB had significantly fewer apoptotic cells (as assessed by TUNEL assay) when compared to control rats. In addition, they exhibited attenuated release of Cyt-C from mitochondria to the cytosol fraction and a reduced activity of caspase-3, indicating that in this animal model the neuroprotective effect of NGB was associated with the inhibition of the mitochondrial-dependent pathway of apoptosis.

## 5. Pharmacological Modulation of NGB Expression

Considering the central role of neuronal apoptosis in diseases of the central nervous system, and available evidence on the protective role of NGB, a significant research effort has recently been focused on the prospect of developing effective therapeutic strategies based on the modulation of NGB-mediated processes (see [54]). Like other proteins, however, native NGB does not cross cell membranes [106]. Thus, its direct administration does not represent a realistic therapeutic strategy and methods to increase endogenous NGB expression have to be devised.

A small number of chemical agents have been shown to induce NGB expression in vitro. They include cobalt and the iron-chelator deferoxamine (DFO) [31]. DFO also enhances hypoxia-inducible factor levels and the heme oxidation product hemin, which, in turn, can stimulate the NGB expression [107,108]. DFO has been clinically used in the treatment of iron intoxication related to thalassemia, but it may induce allergic reactions and toxicity in different organs [109]. Hemin has been employed in the treatment of acute porphyries, but it is unstable and can lead to adverse reactions such as severe phlebitis [110].

The hypoxia inducible factor HIF-1 can enhance NGB levels as well [111]. However, since the NGB promoter lacks HIF-1-binding hypoxia-response elements [112], the effect is likely indirect.

Treatment with vascular endothelial growth factor (VEGF) has been reported to increase NGB expression in cultured cortical neurons [113] with a maximal effect observed at a dose of 20 ng/mL. Since VEGF can induce HIF-1 expression through VEGFR2 receptors, also this agent probably leads to an indirect NGB induction.

Short fatty acids, such as valproic acid and cinnamic acid, were also shown to be NGB inducers in vitro [114]. Valproic acid is a drug used to treat bipolar mood disorders and seizures, while cinnamic acid is a natural fatty acid obtained from cinnamon oil. The concentrations requested to increase NGB levels (0.1 to 1 mM), however, raised serious concerns on possible side effects in vivo [54].

A promising approach involves 17β-estradiol (E2) which was found to upregulate NGB in neuroblastoma cells [115], in mouse primary hippocampal neurons and in primary astrocytes [116] at physiological concentrations (~1 nM). The estrogen receptor β-mediated rapid activation of p38, a member of mitogen activated protein kinase, resulted required for the E2-induced NGB up-regulation, which was pivotal for the neuroprotective effect of E2 against H_2_O_2_-induced apoptosis [116]. Further support to these findings comes from studies showing that E2 can increase NGB expression and ameliorate the delayed neurological deterioration following aneurismal subarachnoid hemorrhage [117]. Moreover, it modulated huntingtin levels in rat tissues and human neuroblastoma cells [118] and reduced paclitaxel-induced cell death in MCF-7 cell clones [119].

A further hormonal regulation of NGB expression has been very recently identified by Toro-Urrego and coworkers [120]. Since testosterone is a hormone that has been shown to confer neuroprotection by activating anti-apoptotic mechanisms [121], this study was aimed at assessing whether testosterone can also exert protection in glial cells. Thus, the action of the hormone on a human astrocyte cell model, the T98G cells exposed to glucose deprivation, was investigated. The results indicated that testosterone improved cell survival and mitochondrial membrane potential and reduced nuclear fragmentation and ROS generation. Interestingly, these effects were accompanied by a significant up-regulation of NGB expression.

In a rat model of Alzheimer’s disease (AD) a new conjugate of ibuprophen and lipoic acid (IBU-LA) was explored as a possible treatment to counteract AD progression [122]. Immunohistochemistry, Western blot and TUNEL analysis showed that IBU-LA administration had the capability to maintain high NGB levels, allowing NGB to perform a significant antiapoptotic role.

## 6. Concluding Remarks

In the CNS, cell death is related with several diseases including cerebrovascular and traumatic events and neurodegenerative disorders. Due to the high energy demand of the nervous tissue, it appears strongly correlated with mitochondrial dysfunction [12]. In this context, of particular interest is the evidence that NGB, an evolutionary highly conserved protein localized in nerve cells, is both physically and functionally related to mitochondrial functions [18,28,33]. In addition, it confers protection to nerve cells both in vitro and in vivo against a wide range of pathological conditions. To explain its action, many investigations were directed to the interactions the globin can establish with the typical gaseous heme-ligands, playing a role in oxygen sensing, ATP production and scavenging of damaging ROS/RNS [12]. These mechanisms provide a significant contribution to the NGB neuroprotective action by reducing the amount of toxic stimuli impacting on the cells. Furthermore, the redox state of the protein is a key factor defining its interaction with Cyt-C released from the mitochondria. In fact, NGB is able to bind Cyt-C and ferrous NGB can reduce the oxidized Cyt-C to its inactive reduced form, leading to an inhibition of the intrinsic pathway of apoptosis [58,87]. In this respect, additional interactions between NGB and other key proteins involved in the apoptotic process [84] could also play a significant role. They include the interaction with VDAC [62] and the inhibition of the dissociation of G_α_ subunits from heterotrimeric G_i/o_ proteins [61,90]. Furthermore, based on bioinformatics analyses, a much wider role for the protein in the control of the apoptotic process has been hypothesized. It involves the possibility that changes in NGB expression could modulate the level of cellular insult required to trigger the apoptotic cascade [58].

Thus, overexpressed NGB represents a critical player to counteract nerve cell death and the search for drugs and therapeutic strategies targeting NGB overexpression can open significant new opportunities to manage a wide number of very impacting neurological disorders.

## Figures and Tables

**Table 1:** CNS areas expressing NGB.

Region	Species	Assay
*Forebrain*		
Neocortex	Mouse	IHC, ISH [20], RT-PCR, WB [21]
	Human	WB [22]
Subventricular zone	Human	WB [22]
Piriform cortex	Mouse	IHC, ISH [20]
Amigdala	Mouse	IHC, ISH [20]
	Human	ISH [13]
Hippocampus	Mouse	RT-PCR, WB [21]
	Human	WB [22], ISH [13]
Caudatus Putamen	Human	WB [22], ISH [13]
Lateral septal nucleus	Mouse	IHC, ISH [20]
Stria terminalis (bed nucleus)	Mouse	IHC, ISH [20]
*Thalamus*		
Medial and lateral habenula	Mouse	IHC, ISH [20]
Subparafascicular nucleus	Mouse	IHC, ISH [20]
Subthalamic nucleus	Human	ISH [13]
*Hypothalamus*		
Whole hypothalamus	Mouse	RT-PCR, WB [21]
Medial preoptic area	Mouse	IHC, ISH [20]
Suprachiasmatic nucleus	Mouse	IHC, ISH [20]
Periventricular nucleus	Mouse	IHC, ISH [20]
Paraventricular nucleus	Mouse	IHC, ISH [20]
Lateral hypothalamus	Mouse	IHC, ISH [20]
Perifornical nucleus	Mouse	IHC, ISH [20]
Posterior nucleus	Mouse	IHC, ISH [20]
Ventromedial nucleus	Mouse	IHC, ISH [20]
Arcuate nucleus	Mouse	IHC, ISH [20]
Ventral tubero-mammillary nucleus	Mouse	IHC, ISH [20]
*Mid- and hindbrain*		
Substantia nigra	Human	WB [22], ISH [13]
Peripeduncular nucleus	Mouse	IHC, ISH [20]
Subparabrachial nucleus	Mouse	IHC, ISH [20]
Superior colliculus	Mouse	IHC, ISH [20]
Peri aqueductal gray	Mouse	IHC, ISH [20]
Laterodorsal tegmental nucleus	Mouse	IHC, ISH [20]
Pedunculopontine tegmental nucleus	Mouse	IHC, ISH [20]
Locus coeruleus	Mouse	IHC, ISH [20]
Area postrema	Mouse	IHC, ISH [20]
Nucleus of the solitary tract	Mouse	IHC, ISH [20]
Spinal trigeminal nucleus	Mouse	IHC, ISH [20]
Medulla oblongata	Human	WB [22], ISH [13]
*Cerebellum*	Human	WB [22], ISH [13], RT-PCR, WB [21]
*Retina*	Murine	IHC, ISH [25], RT-PCR, WB [21]
	Bovine	IHC, ISH [25]

IHC: Immunohistochemistry; ISH: In situ hybridization; RT-PCR: Real time-PCR; WB: Western blot.

## Abbreviations

NGB	Neuroglobin
Cyt-C	Cytochrome *c*
VDAC	Voltage-dependent anion channel
